# Spreading Effect of tDCS in Individuals with Attention-Deficit/Hyperactivity Disorder as Shown by Functional Cortical Networks: A Randomized, Double-Blind, Sham-Controlled Trial

**DOI:** 10.3389/fpsyt.2015.00111

**Published:** 2015-08-04

**Authors:** Camila Cosmo, Cândida Ferreira, José Garcia Vivas Miranda, Raphael Silva do Rosário, Abrahão Fontes Baptista, Pedro Montoya, Eduardo Pondé de Sena

**Affiliations:** ^1^Postgraduate Program, Interactive Process of Organs and Systems, Federal University of Bahia, Salvador, Brazil; ^2^Neuromodulation Center, Spaulding Rehabilitation Hospital, Harvard Medical School, Boston, MA, USA; ^3^Bahia State Department of Health (SESAB), Salvador, Brazil; ^4^Functional Electrostimulation Laboratory, Biomorphology Department, Federal University of Bahia, Salvador, Brazil; ^5^Institute of Physics, Federal University of Bahia, Salvador, Brazil; ^6^Postgraduate Program in Medicine and Human Health, School of Medicine, Federal University of Bahia, Salvador, Brazil; ^7^Research Institute in Health Sciences (IUNICS-IdisPa), University of the Balearic Islands, Palma, Spain

**Keywords:** attention-deficit/hyperactivity disorder, functional cortical networks, transcranial direct current stimulation, spreading effect, dorsolateral prefrontal cortex

## Abstract

**Background:**

Transcranial direct current stimulation (tDCS) is known to modulate spontaneous neural network excitability. The cognitive improvement observed in previous trials raises the potential of this technique as a possible therapeutic tool for use in attention-deficit/hyperactivity disorder (ADHD) population. However, to explore the potential of this technique as a treatment approach, the functional parameters of brain connectivity and the extent of its effects need to be more fully investigated.

**Objective:**

The aim of this study was to investigate a functional cortical network (FCN) model based on electroencephalographic activity for studying the dynamic patterns of brain connectivity modulated by tDCS and the distribution of its effects in individuals with ADHD.

**Methods:**

Sixty ADHD patients participated in a parallel, randomized, double-blind, sham-controlled trial. Individuals underwent a single session of sham or anodal tDCS at 1 mA of current intensity over the left dorsolateral prefrontal cortex for 20 min. The acute effects of stimulation on brain connectivity were assessed using the FCN model based on electroencephalography activity.

**Results:**

Comparing the weighted node degree within groups prior to and following the intervention, a statistically significant difference was found in the electrodes located on the target and correlated areas in the active group (*p* < 0.05), while no statistically significant results were found in the sham group (*p* ≥ 0.05; paired-sample Wilcoxon signed-rank test).

**Conclusion:**

Anodal tDCS increased functional brain connectivity in individuals with ADHD compared to data recorded in the baseline resting state. In addition, although some studies have suggested that the effects of tDCS are selective, the present findings show that its modulatory activity spreads. Further studies need to be performed to investigate the dynamic patterns and physiological mechanisms underlying the modulatory effects of tDCS.

**Trial Registration:**

ClinicalTrials.gov NCT01968512.

## Introduction

Transcranial direct current stimulation (tDCS), a non-invasive brain stimulation technique, is known to modulate spontaneous neural network excitability (1–3). A weak electrical current modifies the neuronal resting membrane potential to increase or decrease cortical activity according to whether the polarity applied is anodal or cathodal (1, 4). The technique is safe, inexpensive, and simple to apply, and this positive profile increases its potential applicability in different neuropsychiatric disorders such as attention-deficit/hyperactivity disorder (ADHD) (5–8).

In a crossover design, Bloch et al. studied the response of 13 ADHD patients to a single session of high-frequency repetitive transcranial magnetic stimulation (rTMS) (9). An increase in attention score was found in the active group compared to the sham group (9). Although up to the present moment, no studies have been conducted on the effect of tDCS in an ADHD population, the cognitive improvement in executive functions such as inhibitory control and attention observed in previous trials reinforces the potential of this technique as a possible therapeutic tool for use in this population (10–14).

To explore the potential of this technique as a treatment approach, it is important to understand the mechanisms involved in its modulation of brain connectivity. Physiological aspects of brain modulation have been examined in studies conducted in healthy volunteers and in individuals with neuropsychiatric conditions (4, 15–18). Nitsche and Paulus investigated the effects of tDCS on the brain using transcranial magnetic stimulation (TMS) as a tool with which to assess cortical excitability according to changes in the motor evoked potentials (MEP) (19). Using the repeated measures technique, those investigators studied 12 healthy participants submitted to tDCS at 1 mA over the left motor cortex (M1), and reported that the amplitudes of the MEP increased, demonstrating an increase in cortical excitability in the stimulated area (19). In a double-blind crossover trial, patients with major depressive disorder were submitted to anodal tDCS over the left dorsolateral prefrontal cortex (DLPFC) for 20 min at 2 mA (20). Theta changes were observed in the medial frontal cortex area, suggesting that the effect of tDCS consisted of indirect rather than direct modulation on the stimulated area, the left DLPFC (20).

Although previous trials have investigated the distribution and physiological mechanisms of the modulatory effects of tDCS (4, 9, 15, 19–23), up to the present moment no studies have been conducted to assess brain connectivity following tDCS in individuals with ADHD. In this present study, the objective was to use a functional cortical network (FCN) model based on electroencephalographic activity to study the dynamic patterns of brain connectivity modulated by tDCS in individuals with ADHD. The hypothesis to be tested was that a single session of anodal tDCS over the left DLPFC at a current intensity of 1 mA for 20 min increases cortical connectivity compared to a sham group in adults with ADHD.

## Methods

### Participants

Sixty individuals (35 males and 25 females) with ADHD (mean age ± SD: 32.2 ± 10.9 years) participated in the present parallel, randomized, double-blind, sham-controlled trial. To be eligible for inclusion in the study, individuals had to have been diagnosed with ADHD as defined in the Diagnostic and Statistical Manual of Mental Disorders, fourth edition, revised (DSM-IV-TR), with diagnosis confirmed through careful assessment by an experienced psychiatrist. Other inclusion criteria were: age 18–65 years and being capable of understanding and signing the informed consent form. The exclusion criteria consisted of the presence of major psychiatric disorders; cognitive impairment; use of psychoactive substances or alcohol abuse in the previous 12 months; or any contraindication to the use of tDCS such as the presence of a metallic implant in the head or an implanted medical device. Cognitive impairment was defined as a score ≤24 in the Mini-Mental State Examination (MMSE) (24, 25). The Mini International Neuropsychiatric Interview Plus (MINI-Plus) and the adult ADHD Self-Report Scale-18 (ASRS-18), a valid scale based on DSM-IV criteria (26, 27), were the instruments used for screening. With respect to the ASRS-18 assessment tool, subjects had to have a minimum score of 18 points.

**Figure 1:**
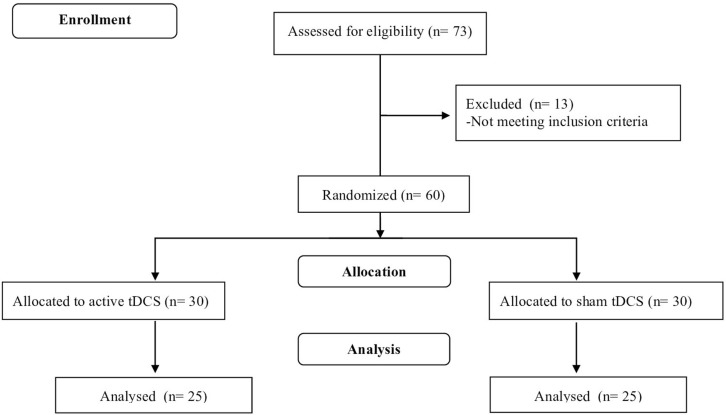
**Study flowchart adapted from CONSORT flow diagram**.

Initially, 73 individuals were recruited through advertisements on the Internet and in social networks, and by e-mails and letters sent to neurologists, psychiatrists, neuropsychiatric societies and associations. Following prescreening interviews conducted by e-mail and over the telephone, 13 subjects were excluded because they failed to meet the inclusion criteria. As shown in Figure 1, the remaining 60 patients composed the final sample.

Participants were randomly allocated to receive active or sham stimulation on a 1:1 basis. A permuted-block method was applied, with gender and age as prognostic variables. Thirty subjects were assigned to each group. An external investigator performed the randomization procedure, which was conducted using a computer-generated list of numbers (central randomization), with individuals being admitted to the study in the order of enrollment, thus guaranteeing the allocation concealment.

The institutional review board (IRB) of the *Maternidade Climério de Oliveira*, Federal University of Bahia approved the study protocol on October 9, 2012 (IRB number: 19311). All the participants gave their written informed consent after receiving detailed verbal and written information about the protocol in accordance with the ethical principles of the Declaration of Helsinki (28). The present study was conducted between May 2013 and April 2014 at the Laboratory of Functional Electrostimulation of the Federal University of Bahia (Salvador, Brazil).

### Procedures

In all cases, the participation of the individuals in the study consisted of a single visit lasting 2 h. Following the screening procedures, individuals were invited to sit in a comfortable chair in a sound-attenuated room before beginning the electroencephalogram (EEG) recording. During the first minute of the EEG recording, the participants were asked to look fixedly at a cross, after which recording took place over 4 min with the participant’s eyes closed, in a resting state. The same EEG recording parameters were applied before and immediately following the interventions.

### Interventions

The tDCS was applied using a Nemesys stimulator (Quark Medical Products, model Nemesys 941, Brazil). A single session of active or sham tDCS was performed in bi-frontal montage, placing the anodal electrode over F3 and the cathodal electrode at F4 (according to the international 10/20 EEG system), corresponding to the left and the right DLPFC area, respectively. A certified researcher administered the tDCS intervention. An electrical current of 1 mA was administered to the scalp through electrodes inserted in 35 cm^2^ saline-soaked sponges (current density 0.029 mA/cm^2^) for 20 min. To avoid discomfort, the current was ramped up over 30 s and ramped down over an equal interval of time at the end of the session (29). For sham stimulation, the current was applied over 30 s and subsequently turned off without the participant’s knowledge to avoid he/she becoming aware of the group to which they had been allocated. This sham procedure mimics the initial perception of stimulation; however, without modulating brain excitability (30–32). To assess the success of the blinding approach, subjects were asked after the intervention whether they had received active or sham tDCS. Neither the participants nor the raters received information regarding which procedure had been applied (whether active or sham).

To assess safety, the participants were asked open-ended questions formulated in accordance with the tDCS adverse events questionnaire (33).

### Functional cortical network model

The FCN model based on EEG activity was applied to assess the acute effects of tDCS (34).

Electroencephalogram was recorded over a 5-min period prior to and following stimulation, totaling 10 min, under two different conditions: 1 min with eyes open, looking fixedly at a cross, followed by 4 min with eyes closed in a resting state. Electroencephalographic recording was performed by applying 32 channels with the Cz electrode as a reference signal, using a BrainNet-BNT device (EMSA Medical Instruments, Brazil). EEG analysis was conducted using the EEGLAB/MATLAB software system (The Mathworks, Inc.). Cup electrodes (Cu/Au) were arranged following the international 10–20 system, with the following additional electrodes: FC3, FC4, CP3, CP4, FT7, FT8, TP7, TP8, and Oz. Electrode-skin impedance was set below 5 kΩ.

To perform the FCN, EEG data was converted from ASCII format to EEGLAB.set and the entire set of data was filtered between 0.5 and 50 Hz. Artifacts were manually removed by visual inspection performed by an experienced investigator blinded to the intervention groups, using continuous artifact rejection. After these procedures, the final files were converted from.set to ASCII to develop the brain networks, using resting EEG data recorded before and after the intervention.

**Figure 2:**
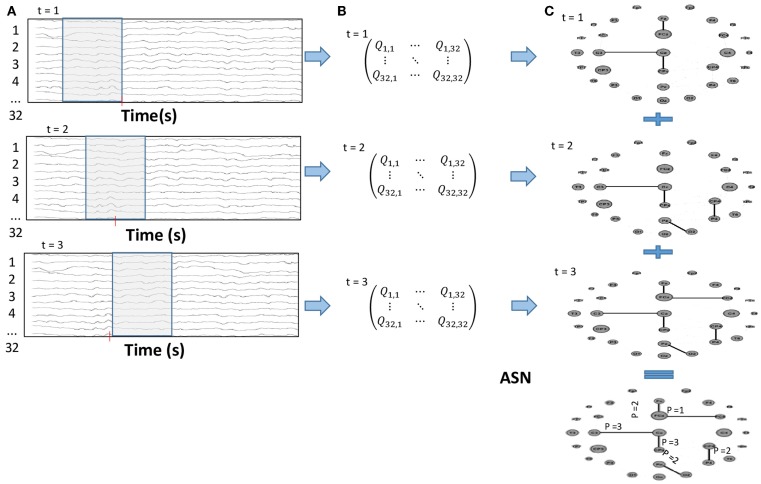
**(A)** Sliding time window over the EEG time series; **(B)** Correlation matrix for each time window; **(C)** After the threshold criterion, the correlation matrices were transformed into a 0 or 1 adjacent matrix, which summed throughout the whole time interval result in the Added Static Network (ASN).

The FCN were constructed using a time-varying graph structure (34, 35) and then analyzing the EEG time series using a sliding time window. A correlation method, Motifs Synchronization, was applied to create a correlation matrix, *Q*_t_ of 32 × 32 for each time window (36). A threshold criterion was used to make a matrix transformation to generate an adjacent matrix *A*_t_ with elements of 1 if the two electrodes were linked or 0 if they were independent. To carry on the weighted network, the Added Static Network (ASN) was used to obtain the sum of all adjacent matrices over the entire time interval, **T**. For the weighted networks, the edge weights represent how many times this link has appeared over the time (Figure 2). Therefore, if {*A*_t_}_t=1,2,…,_**_T_** is the set of adjacent matrices that represents the time-varying graphs {*G*_t_}_t=1,2,…,_**_T_** then ASN is given by:

$$S=\sum_{\text{t}=1}^{\text{T}}A_{\text{ t}}$$

The outcome measure was the weighted node degree, as this parameter describes the network evolution. It corresponds to the number of links presented by a node over time. For the ASN, it is given as the number of times that an electrode was connected to any other in the network over the entire period, **T**, in which a time-varying graph was computed or the node degree was summed over time.

$$kp_{i}=\sum_{\text{t}=1}^{\text{T}}k_{i_{t}}$$

### FCN analysis

FCN analysis was performed using the EEGNET software system. Two resting state networks, pre and post stimuli, were performed for each participant by applying the time-varying graph together with the Motifs Synchronization method. For the purpose of analysis, a sliding time window of 20 points (100 ms), a motif degree of 3, a lag interval of 1, and τ = 3 were taken into consideration. A total of sixty ASN were computed for each intervention group.

### Statistical analysis

The clinical and demographic characteristics were assessed using descriptive statistical procedures such as measures of central tendency and dispersion. At baseline, these parameters were compared between the groups using the chi-square test for categorical variables and one-way analysis of variance (ANOVA) for continuous variables.

The Shapiro–Wilk test was used to assess the normality of data. Parameters of all the electrodes were analyzed for each individual and a weighted node degree was generated for each electrode per group, with *p*-values corrected using the Bonferroni technique. The outcome measure weighted node degree was analyzed applying the non-parametric two-sample Wilcoxon rank-sum (Mann–Whitney) test. Additionally, changes in the outcome were determined as the difference between the weighted node degree after and before the intervention, with the resulting mean for each electrode being compared between groups using the same non-parametric approach. Wilcoxon paired test was used to compare pre- and post-intervention results within groups. Pearson’s chi-square test was conducted to examine the effectiveness of the blinding procedure by assessing the interaction between patients’ impressions of whether or not they had received the tDCS technique and what they had actually been given. Calculation of the sample size was based on previous studies using tDCS (37, 38), for a power of 80% and an alpha error of 0.05. All the analyses were two-tailed. Predicting a dropout rate of 20%, the final sample size was calculated at 60 individuals.

Statistical analyses were performed using the Stata software program, version 13.0 (StataCorp LP, College Station, TX, USA). Statistical significance was determined at 5% and all *p*-values were bidirectional.

## Results

At baseline, no statistically significant differences were found between the active and sham groups for any of the demographic or clinical variables (Table 1). Although all the subjects completed the entire protocol, during the EEG/FCN analysis five participants were removed from each group, since, after artifact removal, fewer than 1200 ms of EEG recording remained. tDCS was well tolerated by all the participants and no adverse events or discomfort were reported.

**Table 1:** **Demographic and clinical characteristics of subjects at baseline**.

	Active group	Sham group
	*n* (30)	*n* (30)
Age (years)^a^	31.83 (11.55)	32.67 (10.37)
Males (%)	56.67	60.0
MMSE^b^	28.77 (1.25)	28.93 (1.20)
Mean duration of disease (years)	21.77	22.90
Types of ADHD (%)^c^
Combined inattentive/hyperactive/impulsive	76.67	70.00
Predominantly inattentive	20.00	23.33
Predominantly hyperactive/impulsive	3.33	6.67

*^a^Age presented as mean ± SD*.

*^b^Mini-mental state examination (MMSE) described as mean ± SD*.

*^c^According to the criteria of the Diagnostic and Statistical Manual of Mental Disorders, fourth edition, revised (DSM-IV-R)*.

*ADHD, attention-deficit/hyperactivity disorder*.

**Figure 3:**
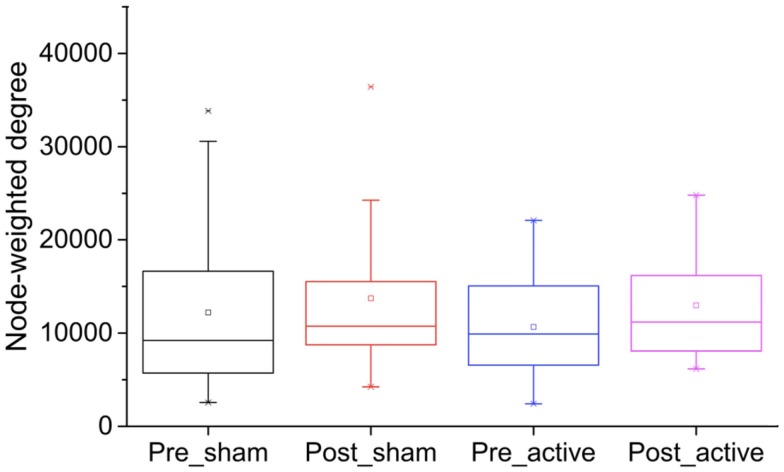
**Box plot representing the weighted node degree by group (active vs. sham) and time (pre vs. post intervention)**. No significant differences were found between the groups before and after the interventions (*p* ≥ 0.05).

Analyses of the weighted node degree prior to the interventions showed no significant differences between the active and sham groups (*U* = 335.00; *p* = 0.86). Results were similar when the weighted node degree was assessed between the groups after the intervention (*U* = 319.00; *p* = 0.92) (Figure 3). Furthermore, no significant differences were found between the groups (active vs. sham) when the changes in the weighted node degree (the difference between the post- and pre-intervention for each electrode) were analyzed (*p* ≥ 0.05; two-sample Wilcoxon rank-sum) (Table S1 in Supplementary Material).

**Figure 4:**
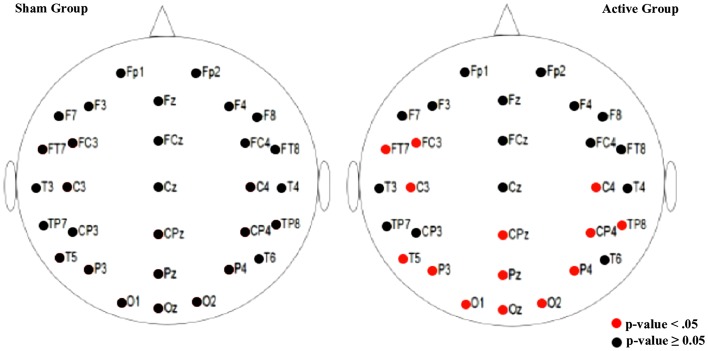
**Illustration of the result by electrode**. The paired-sample Wilcoxon signed-rank test was used to compare the weighted node degree, pre- and post-intervention, within groups. A statistically significant difference was observed in the active group (*p* < 0.05) while no statistically significant differences were found in the sham intervention (*p* ≥ 0.05).

When the weighted node degree was analyzed within the groups prior to and following the interventions, a significant difference was found in the active group with respect to the electrodes located on the stimulated area (left frontal area) as well as on the occipital, left and right temporal, and centroparietal areas (*p* < 0.05). In the sham group, on the other hand, no statistically significant difference was found (*p* ≥ 0.05; Paired-sample Wilcoxon signed-rank test) (Figure 4; Table S2 in Supplementary Material).

Regarding the blinding method, 43.33% of the participants submitted to active stimulation correctly guessed that they were in the active group, while 70% of the individuals in the sham group correctly reported that they had received sham tDCS. However, these differences were not statistically significant (χ^2^ = 1.15; *p* = 0.28).

## Discussion

Analysis of the weighted node degree showed a statistically significant difference in the active tDCS group between the pre- and post-intervention moments, while no statistically significant difference was found in the group submitted to sham stimulation. When the two groups were compared at the post-intervention moment, no differences were detected. These findings suggest that tDCS improves brain connectivity compared to the previous resting state values; however, in the present study, this increase was insufficient to enable differences to be detected between groups.

To the best of our knowledge, this is the first trial to apply a FCN model to examine the dynamic patterns of brain connectivity modulated by tDCS. The FCN model describes brain connectivity based on the correlation between the electrodes over time (34). Although tDCS has been widely studied and applied, its neurophysiological mechanisms remain to be fully clarified (16–19, 39, 40). Therefore, the application of a new physiological measurement approach such as the FCN model presented here is important, since this is a feasible, inexpensive technique compared to neuroimaging or TMS. Furthermore, it allows the spatial changes in cortical connectivity induced by the modulatory activity of tDCS to be followed.

Recent studies have shown the importance of the analysis of temporal dynamics networks in electrophysiological investigation. Chu et al. and Betzel et al. reported the emergence of persistent functional connectivity between pairs of electrodes on a merged network, after summing the time of functional evolution into one single structure (41, 42). Likewise, it has been shown that global measures from evolving networks are different from random networks or those built from surrogate EEG data. Fraiman et al. found significant differences in local network indices for different perceptive behaviors using EEG temporal networks, revealing that cognitive patterns can be identified using time-varying methodology (34). Although all these findings suggest that this type of analysis constitutes a promising tool for the study of the dynamic patterns of cortical activity, the variability in the brain networks between individuals represents a challenge. This may be another explanation for the absence of any significant differences between the intervention groups. In addition, it also emphasizes the importance of paired tests applied in trials using the FCN model, as was done in the present study.

With respect to the paired analysis, an increase in cortical connectivity was found in the active group in the stimulated area, the left frontal area, as well as in the occipital, left and right temporal, and centroparietal areas. Although previous studies emphasized the selectivity of tDCS in the target area under the electrodes (22, 23, 43), the present findings appear to indicate diffuse effects. In a crossover sham-controlled study using EEG to measure the modulatory effects of tDCS, Jacobson et al. detected a localized decrease in theta activity following anodal stimulation over the right inferior frontal gyrus (22). In a trial conducted to investigate language production, Wirth et al. assessed the effects of tDCS using behavioral and electrophysiological parameters. Following anodal tDCS, an increase was found in cortical excitability as shown by a decrease in delta activity in the frontal stimulated area (23).

Despite the evidence reported from earlier studies regarding tDCS modulation of brain excitability in the stimulated area, some studies have proposed that tDCS may modify the neuronal activity of correlated brain areas far from the target region. In agreement with the present findings, Lauro et al. reported a local and diffuse modulation of cortical excitability following anodal stimulation (21). Applying TMS and electroencephalography techniques to examine how the effects of tDCS spread, those investigators demonstrated modulated neuronal connectivity in the target and contralateral hemispheres after 15 min of active stimulation with a current density of 0.08 mA/cm^2^ (21). Modifications in cortical excitability were examined by evaluating cerebral blood flow in a study in which tDCS was applied over M1 (44). Compared to sham tDCS, anodal stimulation increased cerebral blood flow at M1 as well as in the contralateral cortical and subcortical areas, revealing the spread of the effects of tDCS (44). A crossover trial using functional magnetic resonance imaging (fMRI) measured the distribution of cortical excitability after 20 min of tDCS at 2 mA over the left DLPFC (45). Significant cortical activation was detected in the primary area of stimulation and in related brain networks such as the bilateral frontal-parietal and posterior cingulated cortex (45).

The spread of the effects of active stimulation observed in the present study, detected by analyzing the FCN, may be explained by the structural connections between the modulated areas, the left frontal, bilateral centroparietal, and occipital regions. Transcallosal modulation may also support the contralateral findings, as this brain structure is responsible for the majority of axonal connections between the hemispheres (46–48).

Another relevant aspect refers to the effect of electrode location on these results. In the montage used in the present trial, the anodal electrode was placed over the left DLPFC while the cathodal electrode was placed over F4 (in accordance with the international 10/20 EEG system). The findings of the present study may have been the result of anodic activity, since the increase in brain excitability modulated by this stimulation may explain the higher connectivity between brain areas. As shown in Figure 4, excitability was most evident in the left frontal cortex (the stimulated area) and extended to the occipital, left and right temporal and centroparietal areas. In addition, the hyperpolarization promoted by the cathodal stimulation may explain the absence of increased connectivity over the whole right frontal cortex. These findings may support the hypothesis recently raised by Batsikadze et al. that at 1 mA (current density 0.029 mA/cm^2^), anodal tDCS facilitates depolarization, while cathodal tDCS acts as an inhibitory electrode, decreasing cortical excitability, which was not found at a current intensity of 2 mA (49).

Comparison of the groups after the interventions revealed no statistically significant differences, which could be explained by the fact that the application consisted of a single session. Although we understand that multiple sessions of anodal tDCS could increase cortical connectivity, to date this is the first trial to investigate the effects of tDCS in an ADHD population. As this neurodevelopmental disorder involves alterations in brain excitability, with reduced cortical activation in prefrontal areas and increased activity in correlated areas, it was decided to opt for a more conservative approach, using a single session of tDCS to ensure safety while examining its effects in these patients.

As shown in previous studies using EEG and behavioral assessment, tDCS may improve executive functions (10–14, 37, 50, 51), including those affected in patients with ADHD such as inhibitory control and attention, thus suggesting that this technique may represent a possible therapeutic approach for this population. To evaluate the potential of tDCS, our initial focus was on understanding how it affects brain connectivity through the FCN. The aim of the present analysis was therefore to use this mathematical model to investigate the dynamic patterns of brain connectivity modulated by tDCS in patients with ADHD. Notwithstanding, these findings need to be interpreted with caution, since it is not behavioral parameters but, rather, neurophysiological results that are being presented. As mentioned above, this analysis reveals an increase in brain connectivity in the active group when baseline findings prior to tDCS are compared with results after tDCS, showing that this improvement is not limited to the target area but is also seen in correlated regions of the brain. Further studies and analyzes need to be performed to correlate these findings with behavioral assessments to evaluate whether or not higher cortical connectivity might be related to better cognitive performance.

## Conclusion

The present findings suggest increased cortical connectivity, evidenced by a FCN model, in stimulated and related areas following active stimulation over the left DLPFC when compared to the baseline resting state in individuals with ADHD. Although the results of some studies have suggested that the effects of tDCS are selective (22, 23), the present findings and results from other studies show that the spread of the modulatory activity of tDCS, as well as the mechanisms underlying its effects, need to be more fully investigated.

## Author Contributions

CC, AB, and ES conceived and designed the experiments. CC, RR, and ES performed the experiments. CC, CF, JM, RR, AB, PM, and ES analyzed and interpreted the data. CC, JM, and ES drafted the manuscript. CC, CF, JM, RR, AB, PM, and ES performed a critical review of the manuscript. All the authors read and approved the final version of the manuscript.

## Conflict of Interest Statement

The authors declare that the research was conducted in the absence of any commercial or financial relationships that could be construed as a potential conflict of interest.

## Supplementary Material

The Supplementary Material for this article can be found online at http://journal.frontiersin.org/article/10.3389/fpsyt.2015.00111
