# Supplementary material for: Spreading Effect of tDCS in Individuals with Attention-Deficit/Hyperactivity Disorder as Shown by Functional Cortical Networks: A Randomized, Double-Blind, Sham-Controlled Trial
Source: Front Psychiatry. 2015 Aug 4;6:111. doi: 10.3389/fpsyt.2015.00111 (PMC4524049; doi:10.3389/fpsyt.2015.00111)
Supplement: Supplementary file 1 [file Table_1.PDF]

S1 Table. Analysis of the changes in the weighted node degree between intervention groups

Electrode	U-value [a]	p-value [a]
F7	258.00	.30
T3	298.00	.79
T5	210.50	.05
Fp1	269.00	.41
F3	286.00	.62
C3	265.00	.36
P3	291.00	.69
O1	256.00	.28
F8	262.00	.33
T4	324.00	.83
T6	272.00	.44
Fp2	296.00	.76
F4	260.00	.32
C4	251.00	.24
P4	246.50	.20
O2	221.00	.08
Fz	315.00	.97
Cz	251.00	.24
Pz	247.00	.21
Oz	244.00	.19
FT7	225.00	.09
TP7	252.00	.25
CP3	262.00	.34
FC3	282.00	.56
CPz	262.50	.34
FCz	275.00	.48
CP4	239.50	.16
FC4	268.50	.40
TP8	244.00	.19
FT8	292.00	.70

[a] Wilcoxon rank-sum (Mann-Whitney) U test comparing weighted node degree changes (post-intervention minus pre-intervention scores) between groups.
