# Supplementary material for: Spreading Effect of tDCS in Individuals with Attention-Deficit/Hyperactivity Disorder as Shown by Functional Cortical Networks: A Randomized, Double-Blind, Sham-Controlled Trial
Source: Front Psychiatry. 2015 Aug 4;6:111. doi: 10.3389/fpsyt.2015.00111 (PMC4524049; doi:10.3389/fpsyt.2015.00111)
Supplement: Supplementary file 2 [file Table_2.PDF]

S2 Table. Analysis of the weighted node degree within the groups prior to and following the interventions

Electrode	Active Group		Sham Group	
	Z-value [a]	p-value [a]	Z-value [a]	p-value [a]
F7	55.00	.00	97.00	.08
T3	52.00	.00	86.00	.04
T5	25.00	.00	88.50	.05
Fp1	91.00	.06	112.50	.18
F3	92.00	.06	84.00	.03
C3	41.00	.00	53.00	.00
P3	39.00	.00	63.00	.01
O1	36.00	.00	79.00	.02
F8	67.00	.01	132.00	.43
T4	80.00	.03	68.00	.02
T6	68.00	.01	96.00	.08
Fp2	123.00	.30	121.00	.28
F4	84.00	.03	138.00	.52
C4	29.00	.00	88.00	.05
P4	30.00	.00	80.00	.03
O2	16.00	.00	88.00	.05
Fz	77.00	.02	65.00	.01
Cz	53.00	.00	74.00	.02
Pz	32.00	.00	73.00	.01
Oz	6.00	.00	75.00	.02
FT7	41.00	.00	99.00	.15
TP7	52.00	.00	99.00	.09
CP3	63.00	.01	77.00	.02
FC3	49.00	.00	62.00	.01
CPz	49.00	.00	82.00	.03
FCz	67.00	.01	65.00	.01
CP4	30.00	.00	82.00	.03
FC4	71.00	.01	107.00	.14
TP8	48.00	.00	133.00	.44
FT8	79.00	.02	101.00	.10

[a] Paired-sample Wilcoxon signed rank test comparing weighted node degree pre- and post-intervention results within groups.
